# Renal cell carcinoma metastasis to thyroid tumor: a case report and review of the literature

**DOI:** 10.1186/1752-1947-7-265

**Published:** 2013-12-10

**Authors:** Fabio Medas, Pietro Giorgio Calò, Maria Letizia Lai, Massimiliano Tuveri, Giuseppe Pisano, Angelo Nicolosi

**Affiliations:** 1 Department of Surgical Sciences, University of Cagliari, Cagliari, Italy; 2 Department of Cytomorphology, University of Cagliari, Cagliari, Italy; 3 University of Texas, Galveston, Texas, USA

**Keywords:** Follicular adenoma, Renal cell carcinoma, Secondary tumors, Thyroid neoplasm, Tumor-to-tumor metastasis

## Abstract

**Introduction:**

Metastatic neoplasms to the thyroid gland are rare in clinical practice. Clear cell renal carcinoma is the most frequent site of origin of thyroid metastases and represents 12 to 34% of all secondary thyroid tumors. Tumor-to-tumor metastases, in which a thyroid neoplasm is the recipient of a metastasis, are exceedingly rare. We report a case of clear cell renal carcinoma metastatic to a follicular adenoma. This is the tenth case of renal cell carcinoma metastasis to thyroid tumor reported in the literature.

**Case presentation:**

A 62-year-old Caucasian woman with a history of clear cell renal carcinoma was admitted to our institution for multinodular goiter. A histological examination after total thyroidectomy revealed clear cell renal carcinoma metastasis to a thyroid follicular adenoma.

**Conclusions:**

Preoperative diagnosis of secondary thyroid neoplasm is difficult to achieve. The diagnosis of metastatic disease should be taken into account if patients have a history of clear cell renal carcinoma or if there is a multifocal growth pattern and clear cell appearance of the cytoplasm.

## Introduction

Most thyroid gland tumors are primary and include papillary, follicular, medullary and anaplastic carcinomas. Metastatic thyroid gland tumor is an uncommon finding in clinical practice. Common sites of primary tumors that can metastasize to the thyroid gland are lung, breast, kidney, and head and neck [1]. Tumor-to-tumor metastases, in which a thyroid neoplasm is the recipient of a metastasis, are extremely rare [2]; to the best of our knowledge, only nine cases of renal cell carcinoma metastasis to thyroid tumor have been previously reported in the literature (Table 1) [3-7].

**Table 1:** Renal cell carcinoma metastatic to thyroid neoplasm: characteristics of the cases reported in the literature

**Case number**	**Authors**	**Receiving thyroid neoplasm**	**Interval**	**Immunohistochemistry**
1	Bohn *et al.*[3]	Papillary carcinoma	2 Y	THY (−) RCC marker +
2	Rosai *et al. cited by Bohn and Ryska*[3,4]	Follicular adenoma	NA	THY (−)
3	Chacho *et al. cited by Ryska*[4]	Follicular adenoma	Synchronous	Not done
4	Baloch *et al. cited by Bohn and Ryska*[3,4]	Papillary carcinoma, follicular variant	2 Y	THY (−) CK19 (−)
5	Wolf *et al.*[5]	Microfollicular adenoma	NA	Not done
6	Ryska and Cap [4]	Oncocytic carcinoma	13 M	THY (−), vimentin (+), calcitonin (−), EMA (+), CK AE1-AE3 (+)
7	Qian *et al.*[6]	Hürthle adenoma	Synchronous	TTF-1 (−), THY (−), CD10 (+), vimentin (+)
8	Koo *et al. cited by Bohn*[3]	Follicular adenoma	5 Y	TTF-1 (−), THY (−), calcitonin (−). Equivocal CK and CD10
9	Yu *et al.*[7]	Papillary carcinoma	3 Y	THY (−), TTF-1 (−), CD10 (+), vimentin (+), RCC antigen (−)
10	Medas *et al*. (present case)	Microfollicular adenoma	6 Y	TTF-1 (−), THY (−), CD10 (+)

*Abbreviations*: CK, cytokeratin; EMA, epithelial membrane antigen; M, months; NA, not available; RCC, renal cell carcinoma; THY, thyroglobulin; TTF-1, thyroid transcription factor-1; Y, years.

This report cites a case of metastasis of clear cell renal carcinoma (CCRC) to a thyroid follicular adenoma.

## Case presentation

A 62-year-old Caucasian woman with a 20-year history of non-toxic multinodular goiter presented to our hospital for surgical treatment. She had no associated symptoms such as dysphagia, dyspnea, or dysphonia. Six years earlier, the patient had undergone a left nephrectomy for clear cell carcinoma. A physical examination revealed an indolent subhyoid swelling accompanied by a few round nodules. A preoperative neck ultrasound examination showed an enlarged thyroid gland, with a retrosternal left lobe. The whole left lobe was occupied by an isoechoic vascularized macronodule. No suspicious central or lateral neck lymph nodes were found. The indication for surgery was the result of the sonographic features of the lesion, the thyroid volume, and the retrosternal position of the left lobe; for these reasons fine needle aspiration cytology was considered unnecessary. A total thyroidectomy was performed in our department in April 2010. Her parathyroid glands and recurrent nerves were recognized and preserved. The postoperative course was uneventful, and she was discharged on the second postoperative day.

**Figure 1:**
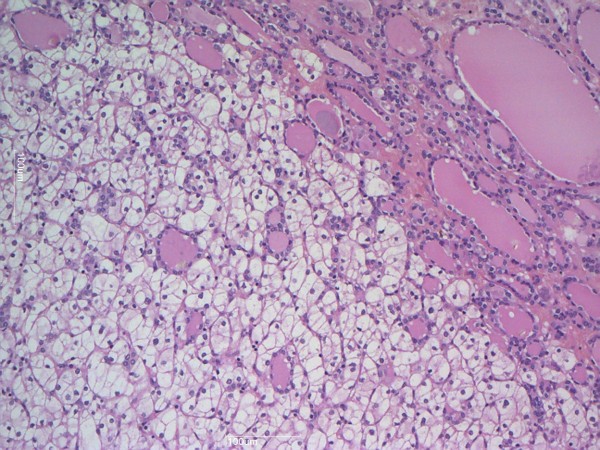
**Hematoxylin and eosin staining demonstrates two different components: microfollicular proliferation and large cells with clear cytoplasm and small and hyperchromic nuclei.** Magnification × 100.

**Figure 2:**
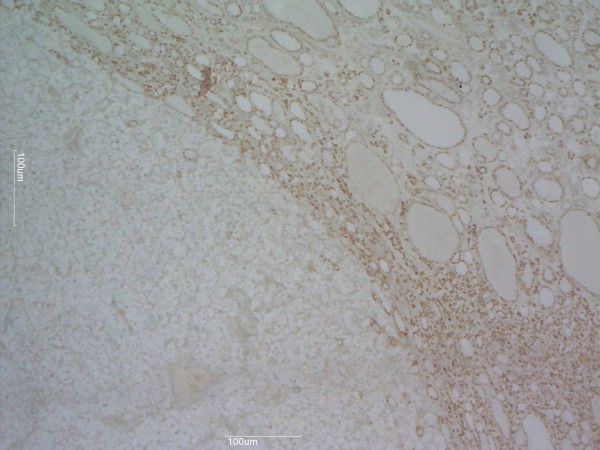
**Immunohistochemical stains.** Thyroid transcription factor-1 is positive in follicular adenoma and negative in metastatic renal cell carcinoma. Magnification × 40.

**Figure 3:**
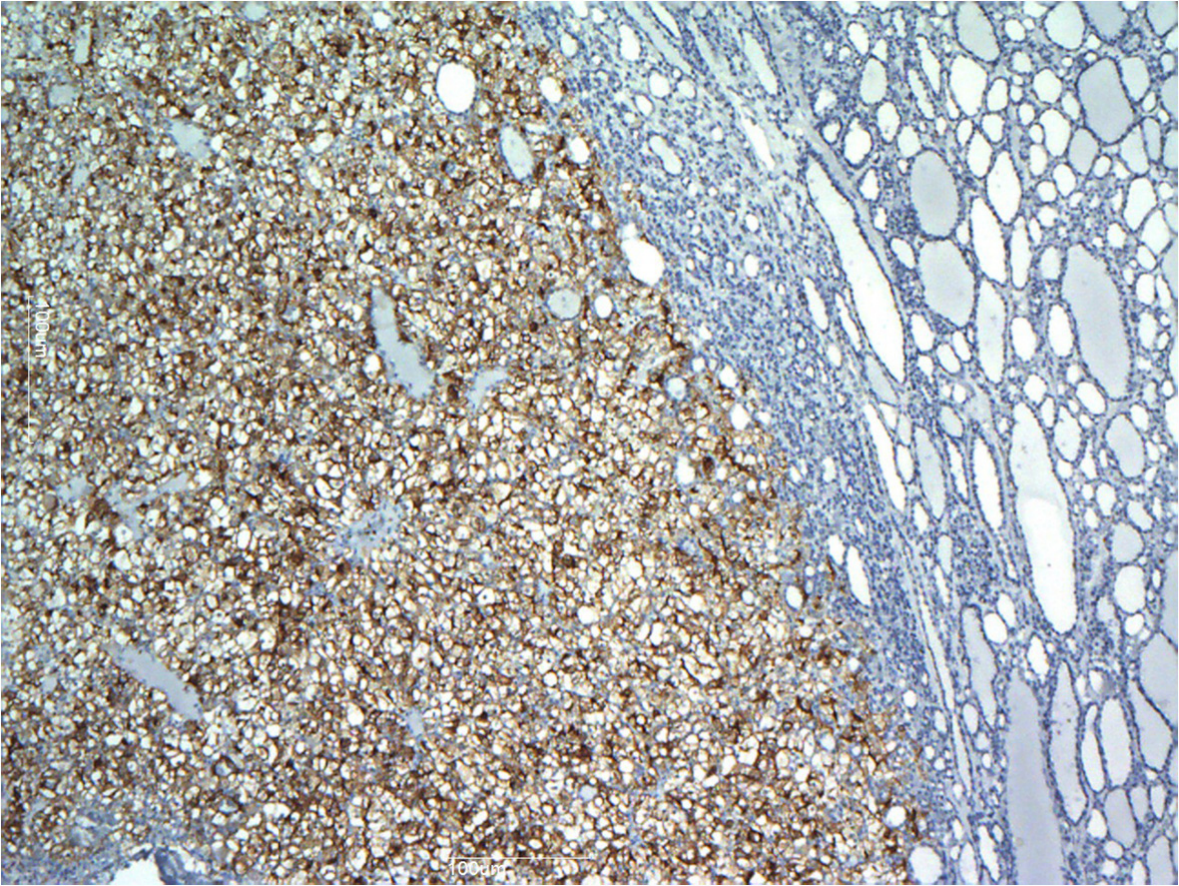
**Immunohistochemical stains.** CD10 is positive in metastatic renal cell carcinoma and negative in follicular proliferation. Magnification × 40.

Upon macroscopic examination, her thyroid was enlarged and multinodular. The left lobe, which was larger than the right one, was occupied by a solid macronodule measuring 53mm; small yellow areas were observed within this nodule. A microscopic examination of the gland revealed thyroid hyperplasia. The left lobe nodule was characterized by microfollicular proliferation with edema and areas of sclerosis and was surrounded by an incomplete fibrous capsule. Inside the nodule, small yellow solid areas were present, and there was a proliferation of large cells with clear cytoplasm and small and hyperchromic nuclei. This second population of tumor cells was well-demarcated from the first (Figure 1). Neoplastic cells were immunonegative for thyroid transcription factor-1 (TTF-1), Hector Battifora mesothelial cell monoclonal antibody-1, and galectin-3 (Figure 2), whereas CD10 was positive (Figure 3). Specimens of the previous renal cell carcinoma were examined, and histopathological features matched the present histology. With these findings, a diagnosis of metastasis of renal cell carcinoma in thyroid follicular adenoma was made.

Postoperative staging demonstrated no other foci of CCRC metastasis.

Three years after her thyroidectomy, no evidence of recurrent CCRC has been found.

## Discussion

Tumor-to-tumor metastasis can be diagnosed when the recipient tumor is a true neoplasm and the donor neoplasm is a true metastasis [2,8]. Growth of a neoplasm into an adjacent tumor or the presence of only tumor emboli within a recipient neoplasm must be excluded. Our case of CCRC metastasis to a thyroid follicular adenoma meets these criteria.

CCRC represents 3 to 4% of all adult malignancies and is the third most frequent urologic cancer [9,10]. CCRC constitutes approximately 85% of all primary renal tumors; its incidence increases with age [9,10]. Nowadays, approximately 40% of kidney neoplasms are diagnosed incidentally due to the widespread use of imaging technologies. Data from the US Surveillance, Epidemiology, and End Results study show that 17% of the patients have metastatic disease at diagnosis. The most frequent sites of metastasis are lung, bone, liver, adrenal gland, contralateral kidney, retroperitoneum, brain, and skin; head and neck metastasis are less frequent, and the thyroid is the site most commonly affected. CCRC recurrence after nephrectomy is highly variable, presenting with metastasis ranging from a few months to several years after the initial diagnosis [9,10].

Metastatic tumors represent 2 to 3% of all thyroid malignancies; [3] the most common primary tumors are skin, breast, lung, kidney, and head and neck. The higher rate of secondary thyroid gland tumors found in autopsy studies, ranging from 5 to 24% [1], suggests that metastatic thyroid lesions are often occult. Metastatic thyroid tumors can represent a first finding of unknown primary tumor (occult primary neoplasm) or a synchronous or metachronous manifestation of known primary tumors.

Some authors have suggested that the thyroid is a common site of metastasis because of its rich blood supply; they proposed that the thyroid gland would be more susceptible to metastatic growth when affected by goiter, neoplasms, or thyroiditis due to metabolic changes that consist of decrements in the oxygen and iodine content [11]. By contrast, other authors have reported that there is no difference in frequency of metastasis in altered thyroid glands versus normal thyroid glands [11].

CCRC metastases to the thyroid gland account for 12 to 34% of all secondary thyroid tumors [1]. Metastatic thyroid tumors may represent the first manifestation of CCRC or a synchronous or metachronous metastasis of a known CCRC. Metastases usually appear as metachronous lesions, often several years after nephrectomy. As in this case, metastatic CCRC may first be misinterpreted as a primary thyroid tumor.

In our case, a secondary thyroid tumor was the first metastatic site during follow-up for CCRC that had been diagnosed 6 years earlier.

A tumor-to-tumor metastasis, in which a thyroid neoplasm is the recipient tumor, was previously described in the literature in only 28 cases [2,12], and ours is the 29th; the most common primary tumor was CCRC with 10 cases (including our case) [3-6,12], followed by lung carcinoma (six cases), breast carcinoma (five cases), colon carcinoma (three cases), and isolated cases of prostate adenocarcinoma, melanoma, pancreatic neuroendocrine carcinoma, Burkitt-like lymphoma, and a malignant phyllodes tumor.

In the 10 cases of CCRC metastasis to thyroid tumor (including this case), the recipient tumor was follicular adenoma in five cases [3-5], papillary carcinoma in three cases [3,12], Hürthle cell adenoma in one case [6], and Hürthle cell carcinoma in one case [4] (Table 1).

Metastases were metachronous in eight cases [3-5,12] and synchronous in two cases [3,6] (Table 1). In one case, the diagnosis was autoptic in a patient with diffuse metastatic disease [5]. Latency from nephrectomy to diagnosis of thyroid metastasis varies from 13 months to 6 years. In the metachronous metastasis group, a thyroid nodule was found during follow-up for CCRC in seven cases with ultrasound examination of the thyroid gland. In one case, pathologic uptake of ^18^F-fluorodeoxyglucose in the thyroid with ^18^F-fluorodeoxyglucose-positron emission tomography was the first finding [5]. In this group, total thyroidectomy was performed in six cases and a loboisthmectomy in one case; in one case, diagnosis was made post-mortem. In the synchronous metastasis group, a total thyroidectomy was performed before nephrectomy for suspicious thyroid nodule, with pathologic findings suggestive for CCRC metastasis; further investigations demonstrated a renal mass, and the patients underwent nephrectomy.

Immunohistochemistry was made in nine cases and stained positive for the CCRC marker and vimentin and negative for TTF-1 and thyroglobulin [2-4,6,12].

Although CCRC metastasis to the thyroid gland can be suspected in patients with a history of renal tumors, preoperative diagnosis of primary versus secondary tumor is difficult. Radiological findings are similar in both cases, with the nodule being a hypoechoic, non-homogeneous, and vascularized mass upon ultrasound examination and “cold” on radioiodine uptake studies. Fine needle aspiration cytology of the lesion can be useful in preoperative diagnosis, suggesting a secondary neoplasm; nevertheless, cytological findings are common in primary and secondary neoplasms, and a metastatic tumor can be easily misinterpreted as a primary tumor [12]. As most authors, we do not perform a core biopsy when cytological results are uncertain. Hence, diagnosis of metastatic CCRC is made with histopathological examination after thyroidectomy. Clinical history of prior malignancies, multifocal growth pattern, sinusoidal pattern of vascularization, and clear cell appearance of the cytoplasm should suggest a secondary thyroid tumor to the pathologist [11,12]. Other tumors with clear cell appearance [11,12], such as paraganglioma [13], differentiated thyroid cancer (papillary, follicular and medullary carcinoma), and lung and salivary gland secondary tumors should be considered in the differential diagnosis [3,11,12]. Immunohistochemistry can be helpful for the differential diagnosis, with metastatic cells of CCRC positive for CD10 and vimentin and negative for thyroglobulin, calcitonin, and TTF-1 [3]. Nonetheless, even in a patient with previous CCRC, a new thyroid mass is more likely to be a primary than a secondary tumor.

Thyroidectomy should be performed in patients with no other metastases; prognosis is good in this group. By contrast, patients with disseminated disease have a poor prognosis and should undergo thyroidectomy only for palliative care of compressive symptoms. Loboisthmectomy may be appropriate if CCRC was preoperatively or intraoperatively suspected and in the presence of a single lesion. In our case, even after taking into account the increased risks in case of reoperation, the preoperative diagnosis of multinodular goiter suggested that total thyroidectomy was the most suitable treatment [14,15].

## Conclusions

Thyroid metastasis should be considered in patients with a thyroid nodule and positive history for CCRC. Preoperative distinction between primary and secondary tumors is difficult to achieve. Immunohistochemistry is a useful method for evaluation of patients with suspected nodules, metastatic cells being negative for thyroglobulin, calcitonin, TTF-1 and positive for CD10 and vimentin. If the thyroid gland is the only site of metastasis, a thyroidectomy must be performed.

## Consent

Written informed consent was obtained from the patient for publication of this case report and any accompanying images. A copy of the written consent is available for review by the Editor-in-Chief of this journal.

## Abbreviations

CCRC: Clear cell renal carcinoma; TTF-1: Thyroid transcription factor-1.

## Competing interests

The authors declare that they have no competing interests.

## Authors’ contributions

FM conceived of the study and drafted the manuscript. PGC participated in the design and coordination of the study. MLL carried out the immunoassays and helped to draft the manuscript. MT participated in the design of the study and helped to draft the manuscript. GP participated in the design of the study and helped to draft the manuscript. AN conceived of the study, and participated in its coordination. All authors read and approved the final manuscript.
